# Condition Monitoring of Induction Machines: Quantitative Analysis and Comparison †

**DOI:** 10.3390/s23021046

**Published:** 2023-01-16

**Authors:** Michele Sintoni, Elena Macrelli, Alberto Bellini, Claudio Bianchini

**Affiliations:** 1 Department of Electrical, Electronic, and Information Engineering “Guglielmo Marconi” (DEI), Alma Mater Studiorum, University of Bologna, 47522 Cesena, Italy; 2 Department of Engineering “Enzo Ferrari” (DIEF), University of Modena and Reggio Emilia, 41125 Modena, Italy

**Keywords:** electric machines, fault diagnosis, wavelet transforms

## Abstract

In this paper, a diagnostic procedure for rotor bar faults in induction motors is presented, based on the Hilbert and discrete wavelet transforms. The method is compared with other procedures with the same data, which are based on time–frequency analysis, frequency analysis and time domain. The results show that this method improves the rotor fault detection in transient conditions. Variable speed drive applications are common in industry. However, traditional condition monitoring methods fail in time-varying conditions or with load oscillations. This method is based on the combined use of the Hilbert and discrete wavelet transforms, which compute the energy in a bandwidth corresponding to the maximum fault signature. Theoretical analysis, numerical simulation and experiments are presented, which confirm the enhanced performance of the proposed method with respect to prior solutions, especially in time-varying conditions. The comparison is based on quantitative analysis that helps in choosing the optimal trade-off between performance and (computational) cost.

## 1. Introduction

Maturity, reliability, ruggedness and versatility make the induction motor one of the most widespread electric machines in industrial applications [1]. Nevertheless, condition monitoring is of primary importance. Early detection of incipient faults is essential to take action in time. A fast, unscheduled maintenance can avoid more harmful consequences in the machine, thus decreasing downtime and, ultimately, reducing financial loss. Motor damage can happen at a mechanical level (bearing faults, air gap eccentricity, shaft bending) or at an electrical level (stator and rotor faults).

Rotor faults, such as bar and end-ring breakage, only account for about 5% of induction machine faults [2], but their detection is of primary importance. Stator design has been the subject of large improvements over the years, and stator fault consequences are such that a machine cannot last more than a few seconds with a fault. On the other hand, rotors still maintain traditional structures, mostly the squirrel cage. Moreover, in case of rotor faults, the machine operation is not restricted and it is still possible to save it from more serious consequences, provided that unscheduled maintenance is carried out as soon as possible. Thus, squirrel-cage rotor faults are the focus of the proposed analysis.

An ideal diagnostic procedure requires online implementation, with minimum impact on machine operations, and should avoid additional sensors or estimation of several quantities. Hence, traditional methods are based on signal processing of electrical quantities, such as stator currents. Signal processing includes frequency domain tools, time domain tools and time–frequency analysis [3,4].

One of the most widespread diagnostic procedures is motor current signature analysis (MCSA). MCSA investigates the signatures of the fault, which are specific components in the stator current spectrum [5]. MCSA fails in time-varying conditions or at lower mechanical loads when slip values are low, because rotor fault signatures depend on machine slip. In the former case, the fault signatures are blurred and spread in a wide frequency bandwidth as large as the speed range, and in the latter case, the fault signature is very close to the fundamental component, related to electrical signal frequency. The amplitude of the fault signature is typically several orders of magnitude lower than the fundamental component. Hence, they can be properly distinguished only with a very high time acquisition window, thus achieving very high-frequency resolution. Another classical frequency domain method is extended park vector approach (EPVA) [6], which is affected by the same drawbacks as MCSA. In [7], these shortcomings were addressed by the combined use of the maximum covariance method for frequency tracking (MCFT) and ZFFT algorithm. Another method is based on the current signal envelope [8] computed by the Hilbert transform [9,10]. This method is very promising, as it moves the fault signatures away from the fundamental. Still, its performances are poor in time-varying conditions.

The performances of methods based on time domain analysis are quite satisfactory even in time-varying conditions. In [11,12], the rotor fault signatures in the stator current were demodulated and the energy of the demodulated signal in a specific bandwidth was used as a fault indicator. However, methods based on time domain are affected by a higher level of noise. Moreover, it is quite difficult to achieve good results with low slip values.

To overcome these limitations, time–frequency approaches were largely investigated; in fact, they are an optimal trade-off between frequency domain and time domain techniques. One of the most-investigated methods is the discrete wavelet transform (DWT). Thanks to DWT decomposition, a signal can be efficiently split into different bandwidths with optimal resolution. In [13,14,15], DWT was used to perform a bandwidth decomposition and to identify the details where the effects of faults show up in stationary conditions. In [16,17,18], DWT was used to process start-up signals, thus allowing the detection of incipient rotor bar faults, even in time-varying conditions. The presence of rotor faults is assessed by checking the time evolution of these details. However, DWT could still be ineffective at low load condition or with variable slip, because fault signature components in the spectrum could be covered by the fundamental component.

This issue can be solved by more complex transforms, e.g., the Dragon transform increases the frequency resolution around the fundamental [19]. However, they entail a higher computational cost, while minimum complexity is a desirable requirement for industrial applications.

The frequency resolution around the fundamental can be increased by combining two methods, e.g., applying DWT to the envelope of stator current. This joint method was only used in [20,21,22,23] for constant speed operations. In [24], it was applied also in time-varying conditions. In this paper, this joint method is further investigated. Specifically, here, the stator signal envelope is computed by means of the Hilbert transform and then processed with DWT, for both stationary and time-varying conditions with some innovative procedures.

All the aforementioned DWT-based methods rely on a qualitative analysis of the time evolution of the details related to rotor faults. In this paper, the diagnostic procedure is based on the energy of the details, not on their time evolution, as in [25]. A robust diagnostic index is obtained using the energy that smooths the effects of non-precise identification of the fault signature. Moreover, the energy is normalized to the value obtained in healthy conditions, thus realizing a differential diagnosis that masks the effect of aging and local noises.

In this paper, the bandwidth where the fault signature component is located as a function of the slip value is identified, as in [26]. In contrast, in the previous literature, all the levels were monitored. In summary, in this paper, the Hilbert transform and DWT are used to select an optimal bandwidth where fault components are located and to compute a robust diagnostic index based on energy. In addition, this method could be applied to the detection of other possible machine faults, such as bearings or stator faults, provided that the frequency signature in the stator current spectrum is known.

A quantitative analysis of all methods was made to assess performances in different operating conditions. The comparison between different methods can be used to select the optimal solution in terms of computational cost and performance.

The paper is organized as follows. In Section 2, the theoretical background of the proposed procedure are presented. In Section 3, the two methods typically used for diagnosis, MCSA and demodulation, are briefly reviewed. In Section 4, the proposed method is presented. Section 5 provides a comparison between MCSA, demodulation, the DWT-based technique of [17] and the proposed method. The comparison is carried out by numerical simulations, performed in stationary and transient conditions. In Section 6, experimental results are presented that provide a validation of the assumptions and of simulation results. Section 7 summaries the results of the different methods, and, finally, Section 8 draws some conclusions.

## 2. Modelling of Induction Machine Rotor Faults

In squirrel-cage induction machines, rotor faults are mainly caused by bar or end-ring breakage. These rotor asymmetries entail electrical signals asymmetries in the machine, where the fault signatures are detectable even at an incipient stage.

Specifically, sideband components at (1±2s)·f appear in the stator current spectrum, where *s* is the slip and *f* is the fundamental component of the supply frequency [27]. Additional harmonics with lower amplitude appear at (1±2ks)·f.

Fault severity can be linked to the amplitude of the sideband components with a simplified relationship [28]:

(1) $$\frac{I_{l}+I_{r}}{I_{f}}≃\frac{b}{B}$$

where $I_{f}$ is the amplitude of fundamental component in stator current spectrum; $I_{l}$ and $I_{r}$ are the amplitude of the left and right sideband components, respectively; *b* is the number of contiguous broken bars and *B* is the total number of rotor bars. Relationship (1) does not include magnetic saturation, magnetic asymmetry or interbar currents. These three phenomena have a significant impact on rotor asymmetry. Hence, this simplified model can lead to false positive/negative fault alarms [3]. Dedicated analysis or special rotor manufacturing can reduce the impact of these phenomena and improve fault diagnosis efficacy.

In order to compare healthy and faulty conditions by means of numerical simulations, an effective model of rotor bar breakage is required. Here, the approach presented in [24] is used, which models rotor asymmetry as an increase in rotor resistance $\Delta R_{r}$. This increase is a function of the number of broken bars *b* and the total number of bars *B* [29]:

(2) $$\Delta R_{r}=\frac{3b}{B-3b}R_{r}$$

The additional resistance affects dynamic model’s coefficients, resulting in sideband components in stator currents.

Here, this motor model was implemented with numerical simulations, and was tested by operating the machine in a open-loop voltage/frequency control.

## 3. Machine Diagnosis through MCSA and Demodulation

### 3.1. MCSA

The classic motor current signature analysis (MCSA) is a frequency analysis method that relies on the amplitude of the spectrum components related to rotor faults. The sum of the amplitudes of the left and the right sideband components is divided by the amplitude of the fundamental component at the supply frequency *f*. Then, the following parameter is obtained:

(3) $$i_{MCSA}=\frac{|I\left(f_{l}\right)|+|I\left(f_{r}\right)|}{\left|I(f)\right|}$$

where |I(f)| is the amplitude of the stator current spectrum at the fundamental component, and $f_{l}=\left(1-2s\right)\cdot f$ and $f_{r}=\left(1+2s\right)\cdot f$ are the left and right sideband components, respectively. The diagnostic index based on MCSA is obtained by normalization:

(4) $$I_{MCSA}=\frac{i_{MCSA,F}}{i_{MCSA,H}}$$

where $i_{MCSA,F}$ and $i_{MCSA,H}$ are the parameters computed by (3) in faulty and healthy conditions, respectively. This normalization allows a comparison between MCSA and the other diagnostic procedures, setting a differential index that smooths the effects of parameter variation.

In stationary conditions, $f_{l}$ and $f_{r}$ are constant, while, in transient, conditions they vary with slip. In transient conditions, the mean value of the sideband components is computed, which is used to compute the diagnostic index. In transient conditions, the fault detection capability of MCSA decreases.

Moreover, MCSA is strongly dependent on the frequency resolution of the acquisition: at low slip, sideband components and the fundamental are quite close. Hence, the fault detection capability decreases with low acquisition time.

### 3.2. Demodulation

This technique is a time domain method, where the stator current i(t) is demodulated in order to obtain the fault signature components [11]. Specifically, the left sideband component is moved to zero frequency by a frequency shift:

(5) $$i_{l}\left(t\right)=i\left(t\right)e^{-j2\pi f_{l}t}$$

The same procedure is applied to the right sideband component:

(6) $$i_{r}\left(t\right)=i\left(t\right)e^{-j2\pi f_{r}t}$$

Then, the amplitude of the fault signature components is the mean value (<>) of the signals $i_{l}\left(t\right)$ and $i_{l}\left(t\right)$. The diagnostic index is obtained as:

(7) $$i_{demod}=\frac{|\langle i_{l}\left(t\right)\rangle \left|+\right|\langle i_{r}\left(t\right)\rangle |}{\left|\langle i(t)\rangle \right|}$$

Finally, as for MCSA, the diagnostics index is computed by normalization:

(8) $$I_{demod}=\frac{i_{demod,F}}{i_{demod,H}}$$

where $i_{demod,F}$ and $i_{demod,H}$ are the parameters computed by (7) in faulty and healthy conditions, respectively.

In transient conditions, the mean values of $f_{l}$ and $f_{r}$ in the time acquisition window are used to perform the demodulation. Being a time domain approach, it is less dependent on the acquisition time than MCSA. However, it is quite sensitive to the noise level.

## 4. Machine Diagnosis by Wavelet Transform

### 4.1. Hilbert and Wavelet Transforms for Signal Processing

The proposed method is based on a combination of the Hilbert and wavelet transforms, in agreement with [20,21].

Sideband components, related to the fault signature, can be seen as modulating signals for the fundamental supply frequency. Hence, the fault signature can be obtained by signal demodulation, implemented by the Hilbert transform that computes the signal envelope. This operation mimics an amplitude demodulation, translating sideband components from (1±2ks)·f to 2sf. Specifically, by processing one of the stator currents i(t) using the Hilbert transform, an analytic signal is obtained, whose amplitude a(t) and displacement ϑ(t) are:

(9) $$\left\{\begin{array}{cc} a\left(t\right)= & \sqrt{{i\left(t\right)}^{2}+{HT\left\{i\left(t\right)\right\}}^{2}}\\ \vartheta \left(t\right)= & \arctan\left(\frac{i\left(t\right)}{HT\left\{i\left(t\right)\right\}}\right) \end{array}\right.$$

where HT{i(t)} is the Hilbert transform of the stator current signal. The square of a(t) represents the stator current signal’s envelope and it can be proven that [25]:

(10) $$\begin{array}{cc} \; & {a\left(t\right)}^{2}\approx \;I_{f}^{2}+\sum_{k}\left(I_{l,k}^{2}+I_{r,k}^{2}\right)+\\ \; & 2I_{f}\sum_{k}\sqrt{I_{l,k}^{2}+I_{r,k}^{2}+2I_{l,k}I_{r,k}\left(2\varphi -\varphi_{l,k}-\varphi_{r,k}\right)}\\ \; & \times \sin\left(2ks\omega t+\varphi^{\prime }\right) \end{array}$$

where $I_{f}$, ω and φ are the amplitude, pulsation and displacement of the fundamental component of stator current, respectively; $I_{l}$, $I_{r,k}$, $\varphi_{l,k}$ and $\varphi_{r,k}$ are the amplitudes and displacement of sideband components for the kth harmonic and $\varphi^{\prime }$ is defined by:

$$\begin{array}{cc} \varphi^{\prime }= & \arctan\left(\frac{u}{v}\right)\\ u= & \left[i_{l,k}\cos\left(\varphi -\varphi_{l,k}\right)+i_{r,k}\cos\left(\varphi -\varphi_{r,k}\right)\right]\\ v= & \left[i_{l,k}\sin\left(\varphi -\varphi_{l,k}\right)-i_{r,k}\sin\left(\varphi -\varphi_{r,k}\right)\right] \end{array}$$

In Relationship (10), the main components are at frequencies 2ksf and their amplitude is related to fault severity. Therefore, by suppressing DC components, fault signature components can be easily identified; the mean value is subtracted from the signal $a{\left(t\right)}^{2}$, obtaining the signal $a^{\prime }{\left(t\right)}^{2}$, which can be used to detect rotor faults.

After processing stator current signal using the Hilbert transform, the wavelet transform comes into play to effectively compute fault signature amplitude. In time-varying conditions, or with high noise or load oscillations, the identification of a single frequency at 2sf in the spectrum is quite complex and can lead to false positive alarms. Moreover, for low slip values, the components related to the fault can be covered by the fundamental. A better approach is to extract the energy of the signal inside a frequency band that includes 2sf. Thus, DWT (discrete wavelet transform) is used to select an optimal bandwidth and compute its energy.

DWT decomposes a signal into a number of time-scale atoms obtained through sequential application of dyadic filters that split the signal into different bandwidths. Thus, for each bandwidth, a ‘set’ of signal samples is obtained. Each of these sets is a time series of coefficients describing the time evolution of the signal in the corresponding frequency band.

**Figure 1:**
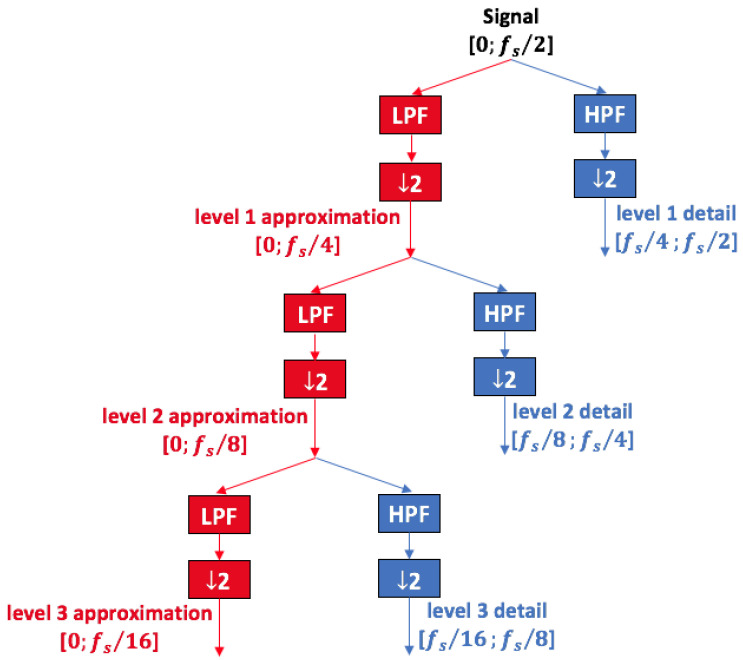
Schematic diagram of DWT decomposition in approximations and details obtained by dyadic filters. At each level the signal is down sampled by a factor of 2, (↓ 2 block). The red color stands for approximation, obtained by low pass filters (LPF block); the blue color stands for detail, obtained by high pass filters (HPF block).

A low-pass filter (LPF) and a high-pass filter (HPF) are applied to the signal in order to compute ‘approximation’ and ‘detail’ atoms, respectively. This operation is repeated at each level *j* on the approximation set, further splitting it. As a consequence, for each decomposition level *j*, approximation $a_{j}$ and detail $d_{j}$ are bandpass signals in the following frequency ranges, respectively:

(11) $$\begin{array}{cc} BW\left(a_{j}\right)= & \left[0\;,\;2^{-(j+1)}f_{s}\right]\\ BW\left(d_{j}\right)= & \left[2^{-(j+1)}f_{s}\;,\;2^{-j}f_{s}\right] \end{array}$$

where $f_{s}$ is the sampling frequency. The decomposition process is outlined in the tree diagram of Figure 1.

Detail atoms include optimal time resolution information for the selected bandwidth. In fact, the detail with higher peaks in the time domain corresponds to the bandwidth that includes the largest share of signal energy. Wavelet transform is subject to the uncertainty principle, but it allows the best time/frequency resolution for the detail’s bandwidth to be obtained. High-frequency details are composed of a high number of samples: high resolution in time domain, and low resolution in frequency domain. In fact, high resolution in frequency domain is not of primary importance for high frequencies. On the other hand, low-frequency details are made of a lower number of samples: frequency resolution is favored over time resolution, which is fundamental to distinguish between different frequency components at low frequency.

**Figure 2:**
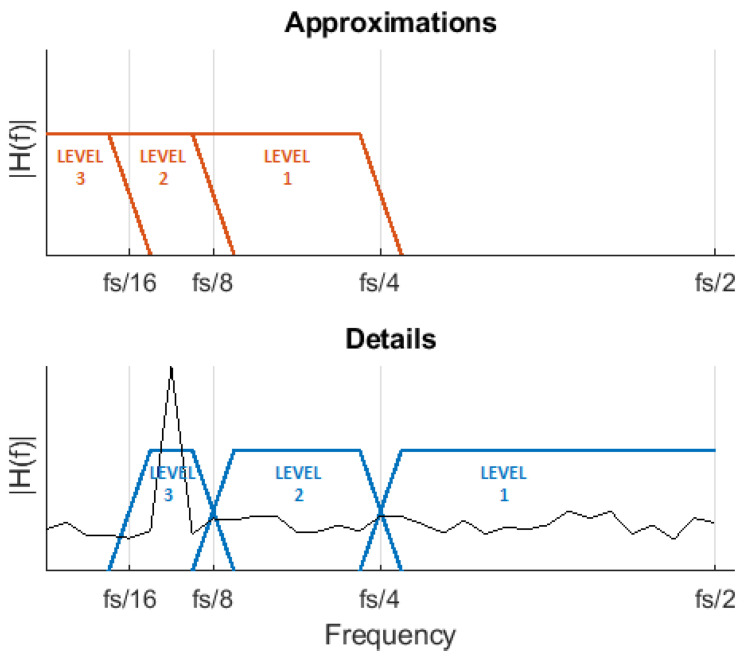
3-level bandwidth decomposition performed by DWT dyadic filters, superimposed on the spectrum of original current signal (black line).

DWT provides optimal accuracy at low frequency and there is no redundant information in the decomposed frequency bands, thus providing a suitable method to estimate the energy associated with the rotor fault. Hence, the proposed method exploits DWT as an efficient time domain algorithm to compute the energy in the frequency bandwidth where the signature component 2sf is localized. Figure 2 reports the spectrum of a current signal (black line) together with the bandwidth of approximations and details, according to (11). In this example, the optimal decomposition is n=3, since the detail for level 3 includes the largest share of energy related to the fault signature.

### 4.2. DWT for Signal Processing

In order to provide a comparison with another wavelet-based technique, an additional diagnostic index based on DWT was computed as in [17]. Here, the stator current signal is not processed by the Hilbert transform. Wavelet decomposition is directly applied to the signal and the index is computed from the energy of the *approximation* atom including the left fault signature component. The optimal number of levels can be computed using the upper boundary relationship for approximation bandwidth: $2^{-\left(n+1\right)}f_{s}<f\Rightarrow \;n=\left\lfloor \log_{2}\left(\frac{f_{s}}{f}\right)\right\rfloor$. Hence, the index is computed similarly to (15):

(12) $$I_{DWT}=\frac{E_{app,F}}{E_{app,H}}$$

with $E_{app,F}$ and $E_{app,H}$ as the energy of the approximation atom in faulty and healthy conditions, respectively.

### 4.3. Proposed Method (HILBERT–DWT)

By selecting the suitable detail from the signal $a^{\prime }{\left(t\right)}^{2}$, the wavelet transform can be successfully used to detect rotor faults. The amplitude of the detail including the frequency 2sf increases with the fault severity (10). Thus, the energy inside this detail bandwidth is chosen as the rotor fault indicator:

(13) $$E_{d}=\frac{1}{N}\sum_{i=0}^{N-1}{\left|d(i)\right|}^{2}$$

where *N* is the number of samples of the detail. In order to select the detail including the frequency 2sf, the optimal decomposition level *n* must be computed using the lower boundary relationship for detail bandwidth (11):

(14) $$\begin{array}{cc} \; & 2^{-\left(n+1\right)}f_{s}<2sf\\ \; & \Rightarrow \;n=\left\lfloor \log_{2}\left(\frac{f_{s}}{2sf}\right)\right\rfloor \end{array}$$

where floor approximation is used to obtain an integer value for *n* [26]. The proposed method can be summarized by the following steps:

- Hilbert transform of a stator line current in order to compute the magnitude of analytical signal a(t). The DC component is suppressed from signal $a{\left(t\right)}^{2}$, obtaining signal $a^{\prime }{\left(t\right)}^{2}$;
- Discrete wavelet transform of signal $a^{\prime }{\left(t\right)}^{2}$ with *n* decomposition levels, where *n* is computed by Relationship (14);
- Computation of energy associated with detail at decomposition level *n* ($E_{d}$), according to (13). $E_{d}$ is continuously monitored in order to check any variation. Variations are most likely associated with a rotor fault event.

The condition monitoring is based on a diagnostic index $I_{H-DWT}$, defined by the ratio between the actual energy of wavelet detail at decomposition level *n* ($E_{F}$) and the energy of wavelet detail in healthy conditions ($E_{H}$) used as a reference:

(15) $$I_{H-DWT}=\frac{E_{F}}{E_{H}}$$

The normalization of (15) makes this method more robust towards parameter variations. It should be noted that Relationship (14) can be used for optimal level selection, provided that the estimation of slip is available. In case of time-varying conditions, the mean value of sf during the acquisition time will be used for the computation.

### 4.4. Drawbacks and Limitations

In order to achieve a robust machine diagnosis with the proposed method, typical shortcomings of the Hilbert transform and DWT must be overcome.

For the Hilbert transform, the first and last samples of the output do not represent the current envelope. These boundary effects must be erased, thus reducing the number of samples with respect to the original current signal. In addition, time-varying conditions cause transient noise in the signal envelope. In agreement with [24], a linear de-trending is applied to the $a^{\prime }{\left(t\right)}^{2}$ signal in order to remove this noise component.

For the wavelet transform, at each decomposition level, the signal must be extended so that the samples are exactly a power of 2. Here, the extension is performed using periodical repetition. Wavelet filter coefficients vary according to different types; Daubechies-44 was used, which has proven to be effective for rotor fault detection [20].

## 5. Simulation Results

The model was simulated and tested in open-loop conditions with constant voltage/frequency speed control. The induction machine model parameters are reported in Table 1. The simulation results were computed with a time acquisition period of $t_{obs}$ = 4 s and a sampling frequency of $f_{s}$ = 10 kHz in different conditions: (1) stationary conditions at constant speed for different values of the supply frequency *f* and requested torque *T*, and (2) transient conditions with a linear ramp of requested torque *T*. Healthy and faulty conditions were simulated according to the bar breakage model of Section 2.

### 5.1. Stationary Conditions

**Figure 3:**
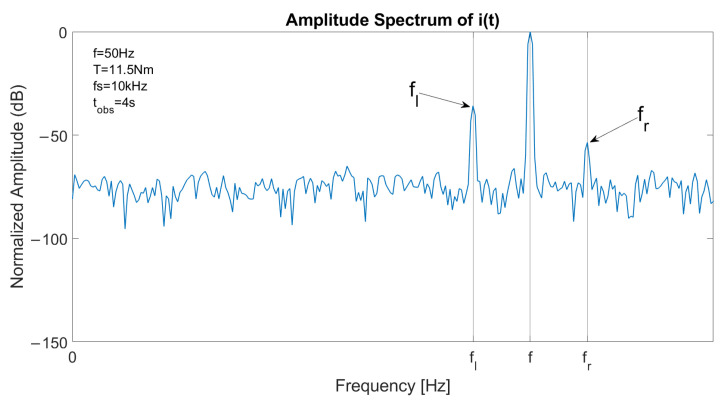
Spectrum of a stator current in case of one broken bar, constant speed at 1500 rpm and slip 6%, with fundamental component located at *f*, left sideband component located at $f_{l}=\left(1-2s\right)\cdot f$ and right sideband component located at $f_{r}=\left(1+2s\right)\cdot f$.

**Figure 4:**
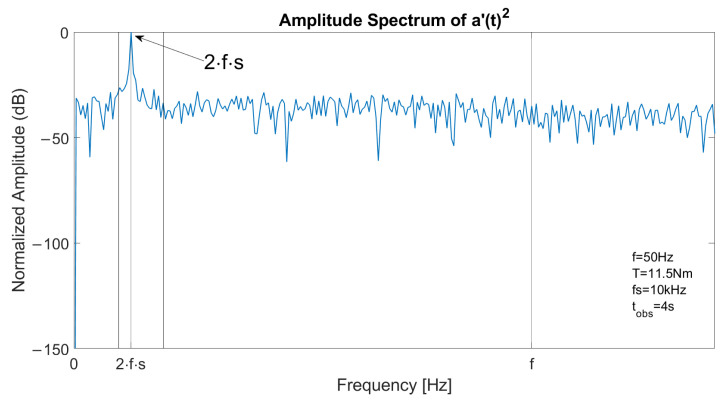
Spectrum of the square of the analytical signal $a^{\prime }{\left(t\right)}^{2}$ of a stator current in case of one broken bar, constant speed at 1500 rpm and slip 6%. The main component is located at 2sf.

The spectrum of one stator current in faulty conditions is reported in Figure 3, with a constant speed at *f* = 50 Hz and *T* = 11.5 Nm. The left sideband is clearly visible at $f_{l}=\left(1-2s\right)\cdot f$ and the right sideband component at $f_{r}=\left(1+2s\right)\cdot f$ has a lower amplitude. Figure 4 reports the spectrum of the signal $a^{\prime }{\left(t\right)}^{2}$ in the same conditions. As the fundamental component is suppressed, the main component is located at 2sf, allowing an accurate fault detection with suitable filtering or energy analysis. The detail of the optimal decomposition level, computed with (14), is confined within the vertical bars shown in the graph. The bandwidth includes 2sf.

**Figure 5:**
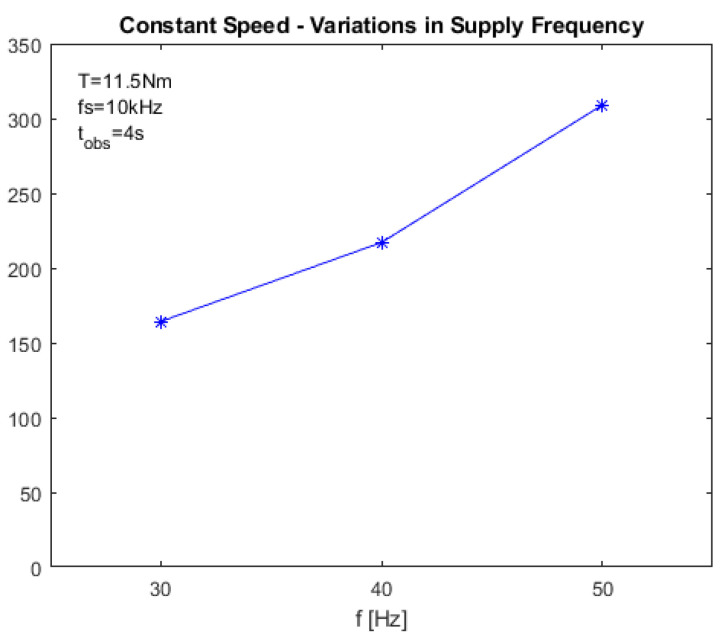
Value of the proposed diagnostic index $I_{H-DWT}$ in stationary conditions as a function of the supply frequency, with T=11.5Nm. The stars state the simulated points, the blue line is the linear interpolation.

**Figure 6:**
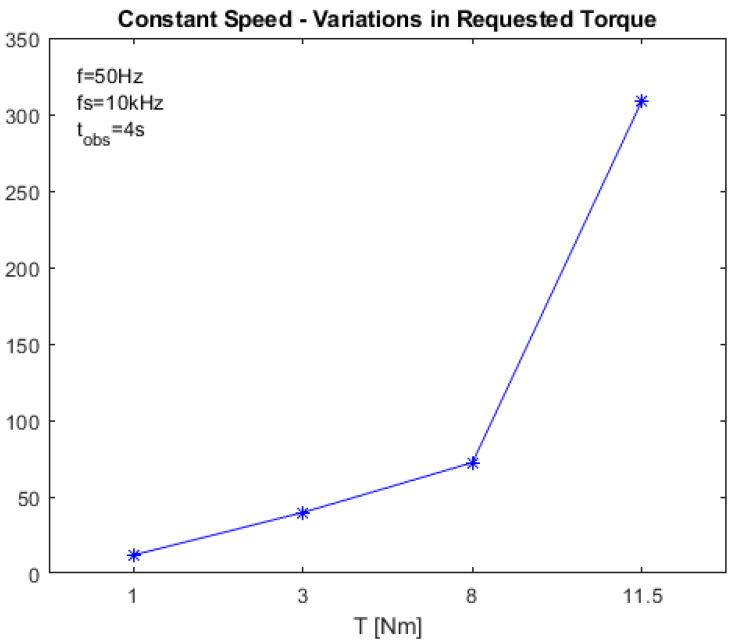
Value of the proposed diagnostic index $I_{H-DWT}$ in stationary conditions as a function of the requested torque, with f=50Hz. The stars state the simulated points, the blue line is the linear interpolation.

The energy of the selected wavelet detail was computed according to (13) in both faulty and healthy conditions. Then, the diagnostic index $I_{w}$ was computed using (15). Figure 5 reports $I_{w}$ as a function of the supply frequency, while Figure 6 reports it as a function of the requested torque.

MCSA and demodulation diagnostic indexes $I_{MCSA}$ and $I_{demod}$ were computed for the same simulations and the results are reported in Table 2 and Table 3. Demodulation and MCSA appear less effective than the proposed approach for fault detection, in particular for low values of frequency and torque.

Simulations were repeated in the same conditions, showing a significant change in the value of the indexes, but for the proposed method. This behavior shows that the diagnostic index is strongly affected by noise. The diagnostic index based on the proposed method, on the other hand, is quite robust and reliable for rotor faults, even if its values decrease for low supply frequency or torque.

### 5.2. Transient Conditions

**Figure 7:**
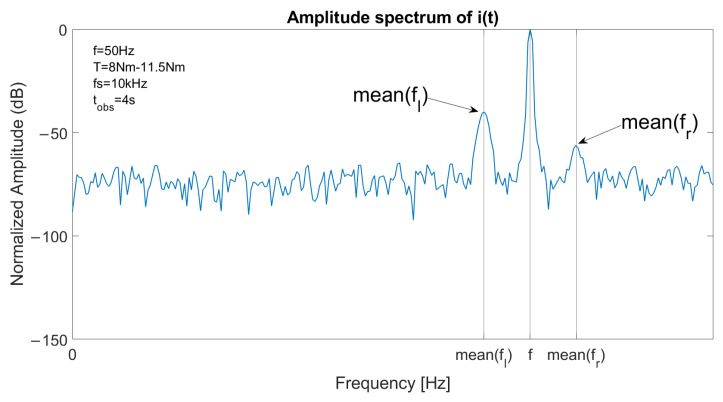
Spectrum of a stator current in case of one broken bar and linear ramp of requested torque from 8 Nm to 11.5 Nm (increasing slip). Fundamental component is located at *f*, left sideband component is located at the mean value of $f_{l}=\left(1-2s\right)\cdot f$ and right sideband component is located at the mean value of $f_{r}=\left(1+2s\right)\cdot f$.

**Figure 8:**
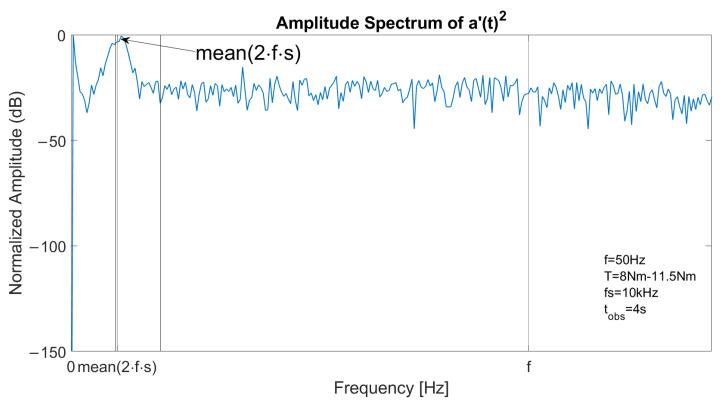
Spectrum of the square of the analytical signal $a^{\prime }{\left(t\right)}^{2}$ of a stator current in case of one broken bar and linear ramp of requested torque from 8 Nm to 11.5 Nm (increasing slip). The main component is located at the mean value of 2sf.

In transient conditions, the spectrum of the stator current is blurred near the sideband components. This effect is visible in (Figure 7), which reports the spectrum of a stator current in faulty conditions with a linear ramp of requested torque from 8 Nm to 11.5 Nm. In this condition, the supply frequency remains constant while the slip varies, thus resulting in a blurred spectrum. Hence, detection with MCSA or demodulation is more difficult. Furthermore, the main component at 2sf in the spectrum of $a^{\prime }{\left(t\right)}^{2}$ is blurred, as shown in Figure 8, which depicts the spectrum of the square of the analytical signal of a stator current in the same conditions. In addition, significant components at low frequencies are visible, related to the slow variation in the slip value.

**Figure 9:**
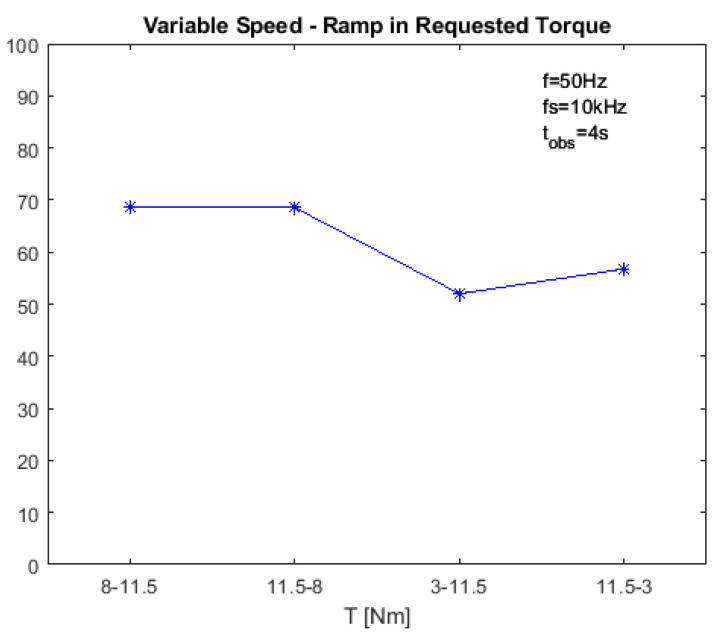
Value of the proposed diagnostic index $I_{H-DWT}$ in transient conditions as a function of the requested torque ramp, with *f* = 50 Hz. The stars state the simulated points, the blue line is the linear interpolation.

With the proposed method, it is possible to overcome this problem by computing the energy in the bandwidth around the fault signature. The optimal decomposition level *n* is computed using (14), with the mean value of the slip during the transient. Figure 9 reports the index $I_{H-DWT}$ as a function of the requested torque ramp, allowing the method’s performances to be assessed. The proposed diagnostic index is still reliable in variable speed conditions, even if its values are slightly lower when low values of torque are involved, as observed in the stationary case.

Table 4 reports a comparison of the indexes computed with the four different methods in transient condition simulations. The results show that fault detection with MCSA or demodulation is unfeasible in cases of variable speed. The higher demodulation index values in cases of torque ramp from 8 Nm to 11.5 Nm are caused by the noise level only. Similarly, the proposed approach outperforms the method based on DWT without the Hilbert transform. This is because the proposed method moves the fault signature component away from the fundamental component, avoiding overlaps in the spectrum in cases of variable frequency or variable load.

## 6. Experimental Results

In order to obtain an experimental validation, the four methods were applied to a series of experimental acquisitions from the testbed realized in [11]. Experiments were performed on a induction machine whose parameters are reported in Table 5, for which two rotors were available: one healthy and the other with one drilled rotor bar. The acquisitions were all performed in stationary conditions, with a time acquisition period of $t_{obs}$ = 10 s, sampling frequency of $f_{s}$ = 10 kHz, supply frequency of *f* = 25 kHz and requested torque of *T* = 3.3 Nm. The load torque level is fairly low, at about one tenth of rated torque.

**Figure 10:**
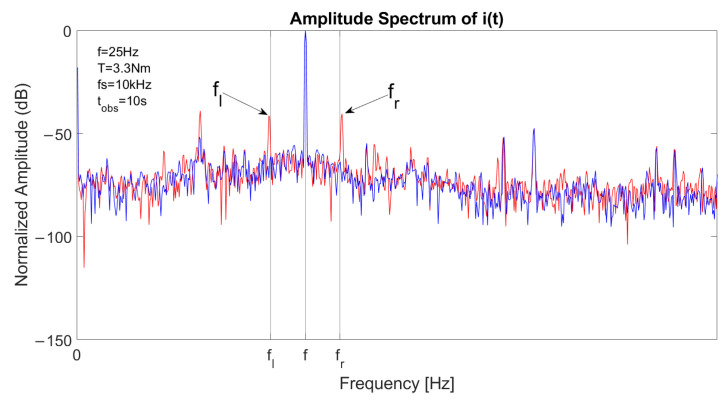
Spectrum of a stator current signal from experimental acquisition in healthy (blue) and faulty (red) conditions, with fundamental component located at *f*, left sideband component located at $f_{l}=\left(1-2s\right)\cdot f$ and right sideband component located at $f_{r}=\left(1+2s\right)\cdot f$.

**Figure 11:**
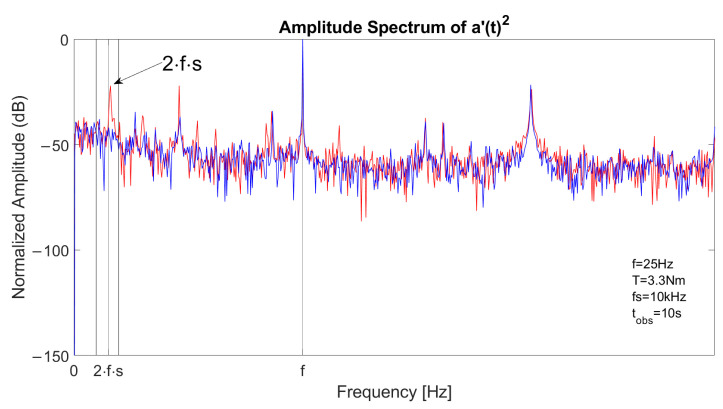
Spectrum of the square of the analytical signal $a^{\prime }{\left(t\right)}^{2}$ from experimental acquisition in healthy (blue) and faulty (red) conditions. The fault signature component is located at 2sf.

The spectrum of a stator current is reported in Figure 10 in both healthy (blue) and faulty (red) conditions. The fundamental component is located at *f*, left sideband component is located at $f_{l}=\left(1-2s\right)\cdot f$ and right sideband component is located at $f_{r}=\left(1+2s\right)\cdot f$. Figure 11 depicts the spectrum of the square of the analytical signal $a^{\prime }{\left(t\right)}^{2}$ with the fault signature component located at 2sf.

Table 6 reports the results for the four methods: index values between two acquisitions in the same conditions do not show significant variation. Therefore, the results can be considered reliable. In stationary conditions, MCSA and demodulation techniques show a satisfying behavior while the performances of the method based only on DWT are low. In any case, the fault detection capability for the proposed method is the highest.

## 7. Comparison of Different Diagnostic Methods

Thanks to numerical simulations and experimental acquisitions, it was possible to compare the efficiency of the proposed technique to three other methods: the classic MCSA (frequency domain approach), the demodulation technique (time domain approach) and another DWT-based approach presented in [17].

Regarding stationary conditions:

- MCSA, demodulation and DWT-only techniques are capable of detecting rotor faults but are highly dependent on the noise level. In fact, the values of the computed indexes vary significantly at each simulation in the same condition.
- The proposed method appears more robust than the other techniques, even if the values of the indexes decrease for low load or low supply frequency conditions.

Regarding transient conditions:

- Fault detection is not possible with MCSA, demodulation or DWT-only techniques. Variation in the indexes’ values is completely random and only due to the noise level.
- Even if the proposed technique is less effective here than in the stationary case, it is still capable of detecting rotor faults. Similar to stationary conditions, its effectiveness decreases for low load conditions.

Experimental results confirm the above items.

In summary, the proposed method appears the best tool for diagnosis because the values of its indexes are less dependent on the variation in the conditions and on the noise level. Its main advantage is the use of a diagnostic index based on the computation of the energy in a bandwidth rather than trying to identify a single frequency. In addition, the demodulation of the signal with the Hilbert transform allows to move the fault signature away from the fundamental component, improving the fault detection capability.

## 8. Conclusions

Preventive and reliable fault detection for induction machines is a topic of increasing importance and interest. State-of-the-art techniques are based on the analysis of the fault signatures in the current spectrum (MCSA) or rely on a time domain approach (demodulation technique). Time–frequency approaches have been also largely investigated, especially methods based on the discrete wavelet transform (DWT). The spectrum components related to faults are blurred during time-varying operation, since they are a function of slip. Thus, these methods fail in transient conditions and are highly dependent on the noise level. Here, a method based on the Hilbert transform and DWT is proposed that significantly improves fault detection accuracy in time-varying conditions. This combination uses the Hilbert transform to demodulate the fault signature components, distancing them from the fundamental, and exploits the high-frequency resolution of DWT at low frequencies to compute an optimal bandwidth with which the fault signature is associated.

The variation in energy of the selected decomposition level is used as a differential diagnostic indicator. Thus, the proposed procedure is less dependent on the noise level or operating conditions than MCSA, the demodulation technique or other methods based on DWT only.

The simulation results show that the proposed method can be effectively used to detect rotor faults in a induction machine with variable speed, using an open loop V/f control. Simulation results were further confirmed by a series of experimental acquisitions. In both cases, comparison with MCSA, demodulation and DWT-only techniques proved that the proposed method is the most robust, because of the large gap between healthy and faulty conditions. A quantitative analysis of all methods was made based on diagnostic indicators. The comparison between the different methods show that the proposed method is a good solution in terms of computational cost and performance.

This technique can be applied not only to the detection of rotor bar faults, but it can also be extended to the detection of other possible machine faults, provided that the frequency signature in the stator current spectrum is known. In addition, thanks to the improved frequency resolution, the proposed method could be of help in the detection of multiple different combined faults.

## Figures and Tables

**Table 1:** Diagnostic indexes at constant speed—experimental acquisitions.

Motor Parameter	Value	Unit
Rated frequency f	50	Hz
Rated voltage V	220	V
Rated power P	2.1	kW
Stator phase resistance $R_{s}$	4.9	Ω
Rotor phase resistance $R_{r}$	3.5	Ω
Stator inductance $L_{s}$	3.56	mH
Rotor inductance $L_{r}$	3.56	mH
Magnetizing inductance M	3.36	mH
Pair poles number P	2	
Rotor bars number $N_{b}$	50	
Viscous friction coefficient F	0	Nm/(rad/s)
Inertia J	0.05	$\mathrm{kg}/m^{2}$

**Table 2:** Diagnostic indexes at constant speed—variations in supply frequency.

	f [Hz]		
	30	40	50
MCSA ($I_{MCSA}$)	0.6992	1.5615	0.4898
Demodulation ($I_{demod}$)	4951.8	34.6	3.1
DWT ($I_{DWT}$)	1.0286	1.115	1.0175
HILBERT–DWT ($I_{H-DWT}$)	164.22	217.07	308.94

**Table 3:** Diagnostic indexes at constant speed—variations in requested torque.

	T [Nm]			
	1	3	8	11.5
MCSA ($I_{MCSA}$)	1.5823	1.1675	0.9024	0.4898
Demodulation ($I_{demod}$)	0.4079	19.258	14.767	3.0821
DWT ($I_{DWT}$)	1.1379	1.0391	1.2821	0.8911
HILBERT–DWT ($I_{H-DWT}$)	11.91	39.74	72.35	308.94

**Table 4:** Diagnostic indexes at variable speed—linear ramp in requested torque.

	T [Nm]			
	8-11	11.5-8	3-11.5	11.5-3
MCSA ($I_{MCSA}$)	0.13955	0.63367	2.771	0.75373
Demodulation ($I_{demod}$)	8.9401	1.013	0.33127	0.4093
DWT ($I_{DWT}$)	0.8854	0.8127	1.0572	0.6502
HILBERT–DWT ($I_{H-DWT}$)	68.755	68.553	52.014	56.765

**Table 5:** Induction motor parameters.

Motor Parameters	Value	Unit
Rated frequency f	50	Hz
Rated voltage V	380	V
Rated power P	7.5	kW
Stator phase resistance $R_{s}$	0.54	Ω
Rotor phase resistance $R_{r}$	0.58	Ω
Stator inductance $L_{s}$	88.4	mH
Rotor inductance $L_{r}$	83.3	mH
Magnetizing inductance M	81.7	mH
Pair poles number P	2	
Rotor bars number $N_{b}$	28	

**Table 6:** Diagnostic indexes at constant speed—experimental acquisitions.

	Acquisition n.1	Acquisition n.2
MCSA ($I_{MCSA}$)	8.5718	10.9707
Demodulation ($I_{demod}$)	5.7109	4.3871
DWT ($I_{DWT}$)	1.0296	1.0097
HILBERT–DWT ($I_{H-DWT}$)	13.9603	14.7246

## Data Availability

Not applicable.
